# Temporal distribution of fishery resources in Payra River: relationship with climatological changes, ecological assessment, and threat assessment

**DOI:** 10.1016/j.heliyon.2022.e10584

**Published:** 2022-09-12

**Authors:** Md. Asikur Rahaman Rahat, Nitai Roy, Md. Rased Khan Manon, Md. Rahamat Ullah, M. Muhsinul Islam, Md. Tareq Rashid, Khandakar Rakibul Hasan, Suprakash Chakma, Md. Arifur Rahman

**Affiliations:** a Department of Fisheries Biology and Genetics, Faculty of Fisheries, Patuakhali Science and Technology University, Dumki, Patuakhali, 8602, Bangladesh; b Department of Biochemistry and Food Analysis, Faculty of Nutrition and Food Science, Patuakhali Science and Technology University, Dumki, Patuakhali, 8602, Bangladesh; c Faculty of Fisheries, Patuakhali Science and Technology University, Dumki, Patuakhali, 8602, Bangladesh; d Riverine Sub-Station, Bangladesh Fisheries Research Institute, Khepupara, Patuakhali, 650, Bangladesh; e Department of Fisheries Technology, Patuakhali Science and Technology University, Patuakhali, 8602, Bangladesh

**Keywords:** Finfish, Shellfish, Biodiversity indices, Ecological pollution, Payra river

## Abstract

The Payra river is one of the large coastal rivers in Bangladesh which supports incredible fish species and has been affected by extensive human disturbance due to huge fishing pressure. The present study provides information about the temporal diversity of finfish and shellfish concerning climatological variables and ecological pollution along with threat assessment in the Payra river, Bangladesh. During the entire study, a total of 61 species including 56 finfish and 5 shellfish species were recorded under 22 families belonging to 11 orders. The order-wise fish species availability showed that the Perciformes (29.49%) was the dominant order based on species richness. Among them, 4 endangered, 6 vulnerable, 4 near threatened, 42 least concern, and 5 data deficient species were found. During the study period, the average Shannon-Weaver diversity index value was (3.33 ± 0.12) indicating a good spread of fish population in the Payra river. Average Margalef richness index value was found (7.60 ± 0.32), Pielou's evenness index (0.48 ± 0.05), and Simpson dominance index (0.93 ± 0.02) in the Payra river. The dominance and Richness index value indicates a clear water environment with slight pollution in the Payra river. Ten different kinds of fishing gears were identified under 3 major groups including 5 nets, 3 hooks and lines, and 2 traps. Canonical correspondence analysis ordination plot showed that rainfall was the most influencing driving force among the meteorological parameters. The cluster analysis based on the Bray-Curtis similarity matrix showed that the winter season formed a separate cluster. In the recapitulation, the Payra river is a highly productive system that provides a favorable environment for a large variety of finfish and shellfish species assemblages. The findings of the conducted study are expected to be helpful for the respective researchers, policymakers, managers, and conservationists for the sustainable management of this water body and the interconnected surrounding neighboring countries.

## 1 Introduction

Bangladesh is endowed with huge and diverse fishing resources as a result of its extensive riverine network like the Ganges (Padma), Brahmaputra (Jamuna), and Meghna Rivers. Estuaries are dynamic habitats marked by considerable swings in environmental conditions because they are the meeting location of freshwater from rivers and saltwater from the sea (James et al., 2007). Estuaries are utilized as nursery grounds by many marine creatures, and they spend part of their life cycle in both fresh and saltwater. Coastal rivers are an important natural ecosystem in Bangladesh (Mahmood et al., 1978), as they provide natural spawning grounds and nursery grounds for many commercially important species of aquatic biota, particularly hilsa (*Tenualosa ilisha*), and they account for a significant portion of the country's fisheries production. As a means of livelihood, more than 11% of Bangladesh's entire population is employed in this sector, both full-time and part-time (DoF, 2018). Overexploitation, siltation, industrial pollution, use of synthetic monofilament nets, overdosing of fertilizers and insecticides in agricultural lands, and ecological disturbances are all contributing to a decline in fish output (Hossain et al., 2012; Rahman et al., 2019a, Rahman et al., 2019b). Changing land use, modifying river flow regimes, riparian and physical habitat loss, water pollution, alien species invasions, and heavy exploitation of fish stocks are only a few of the anthropogenic disturbances that have a significant impact on riverine ecosystems and fisheries (Arthington et al., 2004). As a result, substantially more freshwater species than terrestrial or marine species in the same taxonomic groups are threatened or endangered (Angermeier and Winston, 2015). Fish is important from an ecological standpoint not only because of its economic importance but also because it is sensitive to environmental changes and reflects a wide range of tolerance at the community level (Pielou, 1966). As a result, fish assemblages have been widely employed as biological indicators to quantify and evaluate the amount of river and stream degradation and health (Vijaylaxmi et al., 2010). The Payra river is a body of flowing water that flows to a lower level in a channel on land in Bangladesh, eventually falling into the Bay of Bengal as the Burishwar river. This river has a unique aquatic habitat with a wide range of plants, fish, and other biological types (Islam et al., 2015). Throughout the year, the river has a somewhat turbulent water flow. It is one of the most important coastal rivers in Bangladesh's hilsha migration route and the small hilsha (Jhatka) is most plentiful in this river from January to March (DoF, 2005). The river, which was previously a shelter for brood fishes, has now become rather unsafe due to anthropological factors. According to locals, the diversity of fish species in the Payra river is gradually dwindling due to overfishing, the use of harmful fishing gear (Set bag net), and other issues. Biodiversity is frequently used to assess the state of a biological system's health (Vyas et al., 2012). Previously, Islam et al. (2015) studied the Payra river's fish composition. They did not, however, work on the fish-environment interaction. The current study aimed to determine the river's finfish and shellfish diversity and temporal distribution, as well as the relationship between fish and physicochemical and environmental changes, as well as the level of environmental contamination in Payra. The basic information gathered during this study will be useful in developing and managing the Payra river's development and management program.

## 2 Materials and methods

### 2.1 Ethical approval

The Animal Welfare and Ethical Committee of Patuakhali Science and Technology University, Patuakhali, Bangladesh, oversaw the experimental methodology and guidelines and approved the research and the use of animals in the experiment.

### 2.2 Study area

**Figure 1:**
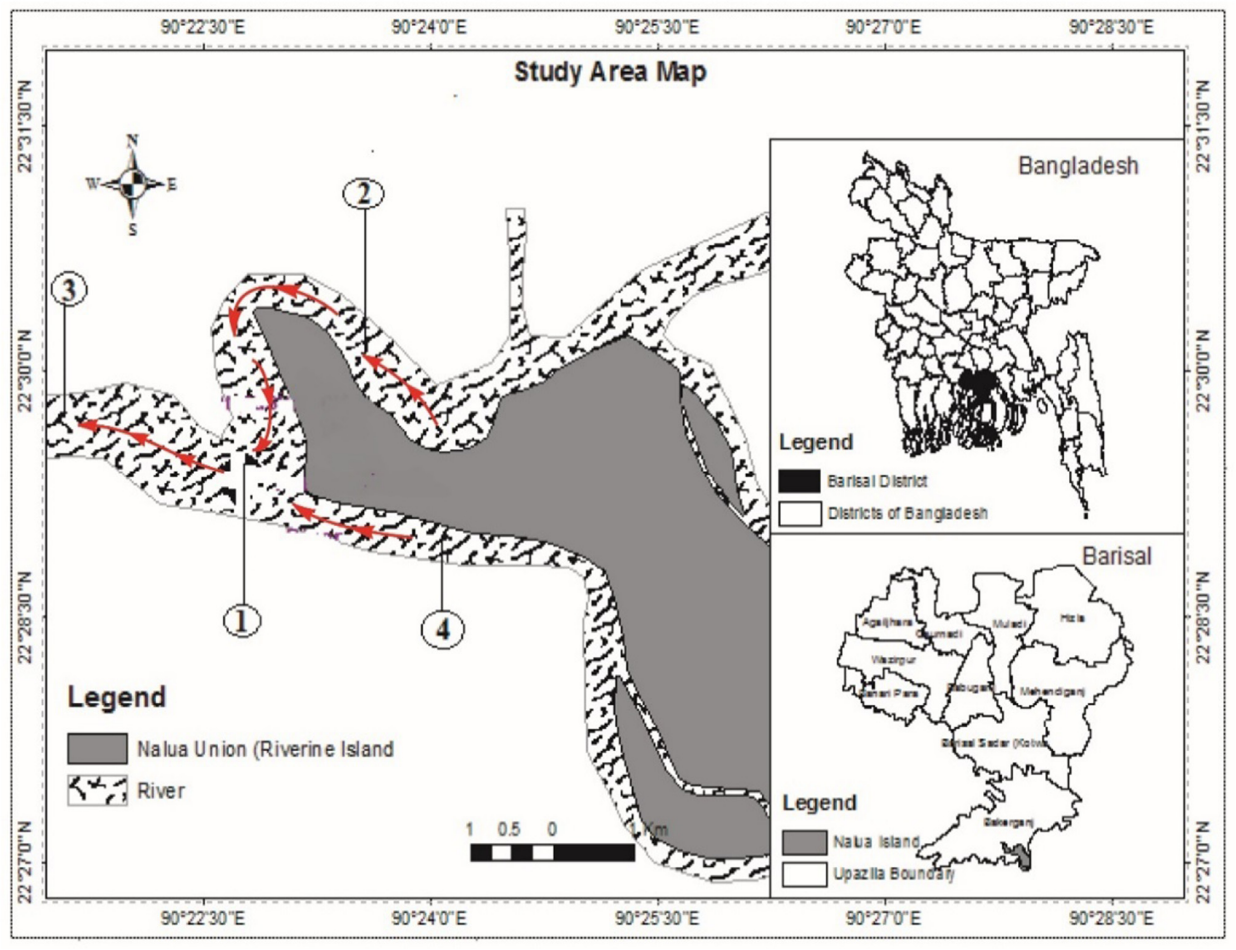
The map shows the reference to the record and geographical location of the study area in Payra river, Patuakhali. The red arrows on the map indicate the direction of water flow in the Payra river system. 1) Karkhana river (Pandab point)- Latitude (22° 29′40.31″N) and Longitude (90° 21′23.10″E); 2) Karkhana river- Latitude (22° 29′18.23″N) and Longitude (90° 22′54.39″E); 3) Khairabad river- Latitude (22° 30′34.52″N) and Longitude (90° 21′24.88″E); and 4) Bighai river- Latitude (22° 48′45.25″N) and Longitude (90° 21′14.17″E).

The Pandav point is the confluence of the Pangasia, Tetulia, and Payra rivers. The Payra river has a large water circulation area. The current study was conducted monthly in the Payra river at Pandav point in the Patuakhali district of the Barisal division from July 2018 to June 2019 (Figure 1).

### 2.3 Finfish and shellfish data collection

We worked with both primary and secondary data in this investigation. Primary data was gathered from local fishermen in the area next to our study area who rely on fishing for a living. Sampling was done on monthly basis based on questionnaire interviews with fishers and a market survey of the adjacent landing center of Pandav point, Payra river area for fish diversity assessment. Other data based on the previous observation was used as a secondary data source to determine the trend of biodiversity loss. The species-to-species data was recorded as species individuals/kg fish and photographs and live fish were taken wherever appropriate for identification based on FishBase (2019). The sampling was done encompassing four seasons: monsoon (July–September), post-monsoon (October–November), winter (December–February), and summer (March–June).

### 2.4 Biodiversity analysis

In the present investigation, the Shannon-Weaver diversity index (H'), Margalef species richness index (d), Pielou's evenness index (J'), and Simpson dominance index (c) were employed to evaluate fishery resources diversity and pollution levels (Margalef, 1968; Pielou, 1966; Shannon and Weaver, 1949; Simpson, 1949) according to the following equations:

$$Shannon-Weaver\;diversity\;index\;\left(H^{\prime }\right)=\;Sum\;\left[\;pi\;x\;log\;\left(pi\right)\right]$$

where, H' = Shannon-Weaver index; Pi = ni/N; ni = no. of individuals of a species; and N = Total number of individuals.

Margalefspeciesrichness(d)=(S−1)/log(N)

where, S = Total species and N = Total individuals.

$$Pielou^{\prime }s\;evenness\;index\;\left(J^{\prime }\right)\;=\;H\;\left(s\right)/H\;\left(max\right)$$

where, H(s) = Shannon-Weaver information function and H (max.) = Theoretical maximum value of H(s).

$$\text{Simpson ​dominance ​index ​}\left(c\right)=\overset{s}{\underset{i=1}{\sum }}{\left(ni/N\right)}^{2}$$

where, ni = number of individuals in ‘each’ species, N = total number of individuals, and S = total number of species.

Moreover, the Shannon-Weaver diversity index (H') and Margalef species richness index (d) value was used to determine the ecological state of the Payra river (Lad, 2015; Staub et al., 1970).

### 2.5 Canonical correspondence analysis (CCA)

The association between the fish and shellfish species assemblage and environmental variables was revealed using Canonical Correspondence Analysis (Marshall and Elliott, 1998). The CCA biplot of the ten most dominant fishery species was used to build the relation between environmental and fish and shellfish species assemblage. For the selection of fish species to compare with environmental characteristics, the method of Rahman et al. (2018) was applied with minor modifications.

### 2.6 Threat assessment

**Figure 2:**
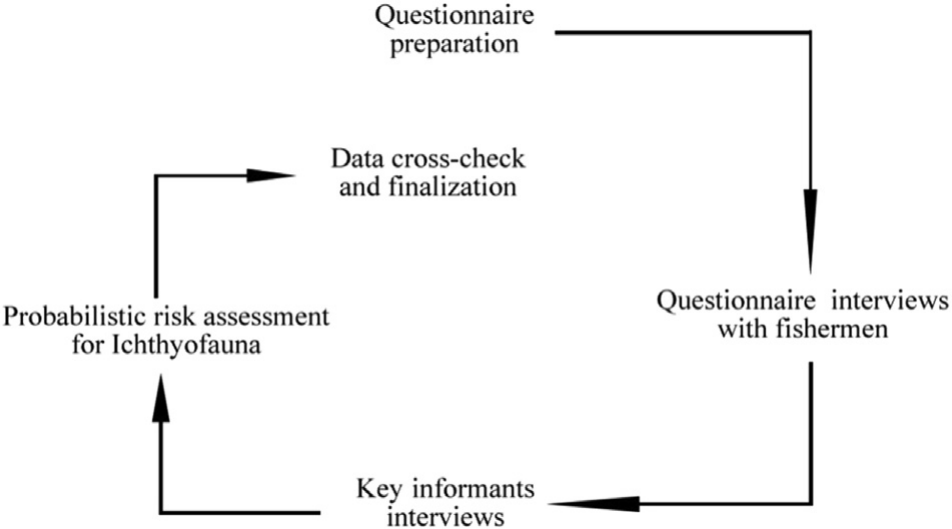
Data collection methods from fishermen to trace probabilistic risk factors and decision-making after validation.

Chemical use, land run-off, and harmful fishing gears employed in the research region were all taken into account while assessing hazards to the Payra river. Thirty key informant interviews were also performed for comprehending the undergoing threat assessments in the study area covering all the sampling sites (Figure 2).

### 2.7 Collection of meteorological parameters

The meteorological data of rainfall (mm), air temperature (**°**C), photoperiod (hrs./Day), and relative humidity (%) were collected from Bangladesh Meteorological Department (BMD), Patuakhali station.

### 2.8 Statistical analysis

A one-way analysis of variance (ANOVA) was employed using the SPSS software version V25.0 (Statistical Package for Social Sciences) for the ANOVA test to see if there was a significant variation in environmental variables and diversity indices between seasons. A Games-Howell nonparametric post hoc analytic approach was used to compare all possible group differences. The software PAST (Paleontological Statistics) version 3.0 was used to perform all of the multivariate analyses like biodiversity indices and cluster analysis. The association between species assemblage composition and environmental variables was investigated using Canonical correspondence analysis (CCA) using PAST (version 3.0) after minor modification of Rahman et al. (2018). ArcGIS 10.0 software was used to map the study region and depict study/sampling locations using a global positioning system (GPS).

## 3 Results and discussion

### 3.1 Environmental parameters of the investigated area

**Figure 3:**
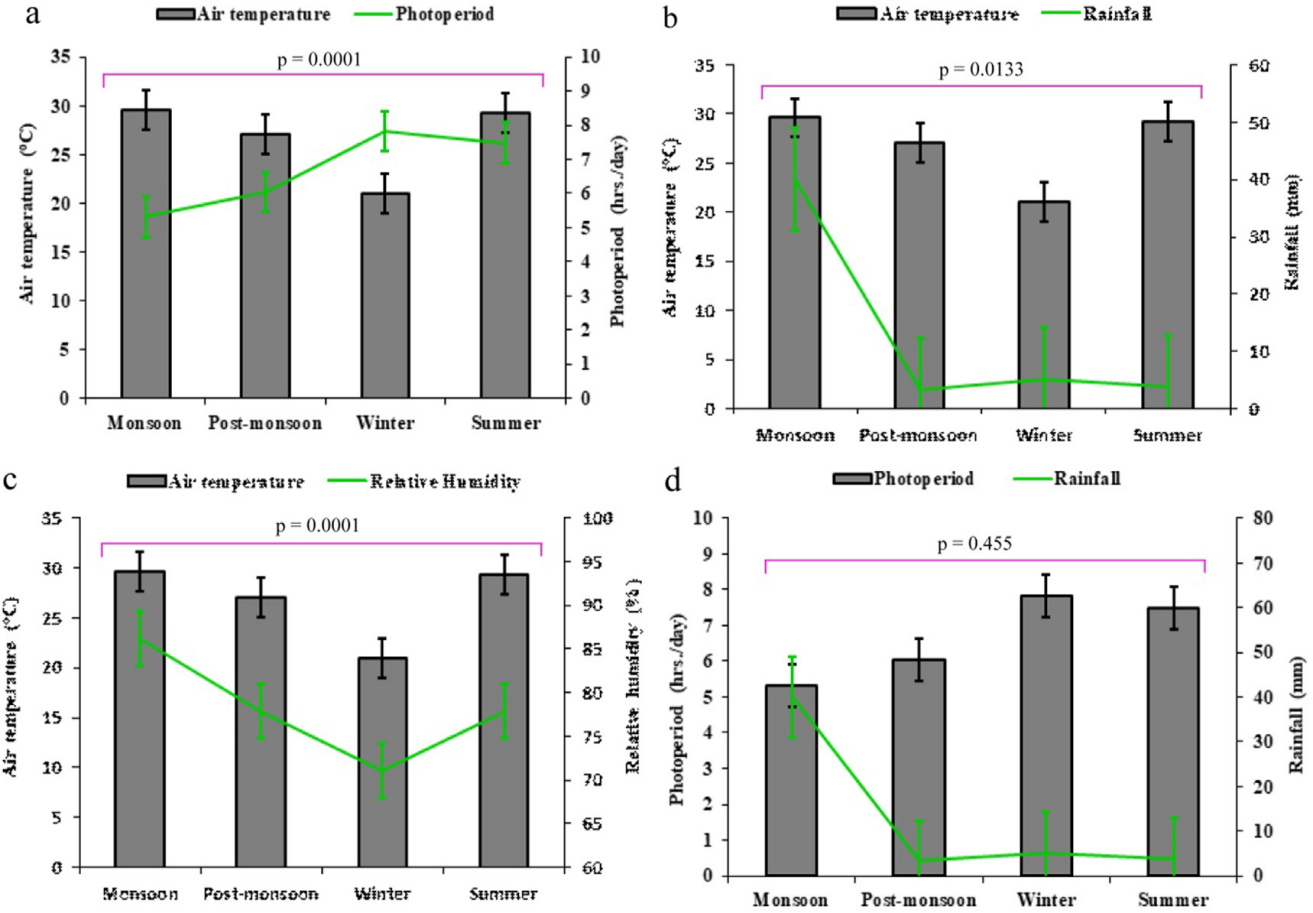
Relationships between climatological parameters during the study period a) Relationship between air temperature (°C) and photoperiod (hrs./day); b) air temperature (°C) and rainfall (mm); c) air temperature (°C) and relative humidity (%); and c) photoperiod (hrs./day) and rainfall (mm). The *p*-value indicates significance at *p* < 0.05 (Two-way ANOVA).

Fish species in open water bodies that are completely reliant on natural feeds (e.g., phytoplankton and zooplankton) are highly sensitive and respond quickly to environmental changes. Environmental characteristics were employed in this study to assess the impact on finfish and shellfish diversity. Table 1 shows the major four environmental characteristics that have been observed to influence the fish assemblage in the Payra river. The maximum atmospheric temperature was recorded at 29.61 ± 0.45 in monsoon and a minimum of 21.01 ± 1.46 in winter with an average value of 26.93 ± 3.95. Besides uppermost photoperiod (hrs./day) 7.82 ± 0.56 was measured in winter and the lowermost 5.31 ± 0.95 in monsoon with an average value of 6.78 ± 1.48. On the other hand, the highest rainfall (mm) of 40.06 ± 53.59 was recorded in monsoon and a minimum of 3.41 ± 4.35 in post-monsoon with a mean value of 13.18 ± 28.39. In addition, higher relative humidity (%) of 86.25 ± 2.63 was recorded in the monsoon and a minimum of 71.00 ± 2.81 in winter with an average value of 78.25 ± 6.46. All climatological variables showed significant differences (*p* < 0.05) between seasons and relationships between climatological parameters are shown in Figure 3. Environmental factors affecting fish communities at local and regional sizes are well understood (Angermeier and Winston, 2015; Cunico et al., 2012; Rowe et al., 2009). Regional factors such as rainfall, humidity, and air temperature, account for some of the differences (Bhatt et al., 2012; Buisson et al., 2008; Dole-Olivier et al., 2009; Ostrand and Wilde, 2001). As a result, our findings back up earlier research that has found that local and regional factors play a role in determining diversification patterns (Jackson et al., 2001; Rathert et al., 1999).

**Table 1:** The climatological parameter's value was collected from Bangladesh Meteorological Department (BMD), Patuakhali station for the study area in different seasons.

Climatological parameters	Monsoon	Post-monsoon	Winter	Summer	*p*-value
Air temperature (°C)	29.61^ab^ ± 0.45	27.06^ac^ ± 2.81	21.01^d^ ± 1.46	29.28^bc^ ± 1.83	0.001
Photoperiod (hrs./day)	5.31^cd^ ± 0.95	6.04^ac^ ± 0.35	7.82^ab^ ± 0.56	7.49^bc^ ± 1.70	0.093
Rainfall (mm)	40.06 ± 53.59	3.41 ± 4.35	5.29 ± 9.16	3.84 ± 3.71	0.342
Relative humidity (%)	86.25^a^ ± 2.63	77.89^bc^ ± 3.03	71.00^d^ ± 2.81	77.88^c^ ± 4.83	0.007

The *p*-value indicates significance at *p* < 0.05 (One-way ANOVA). Value with no superscripts indicates no significance.

### 3.2 Finfish and shelfish diversity

**Figure 4:**
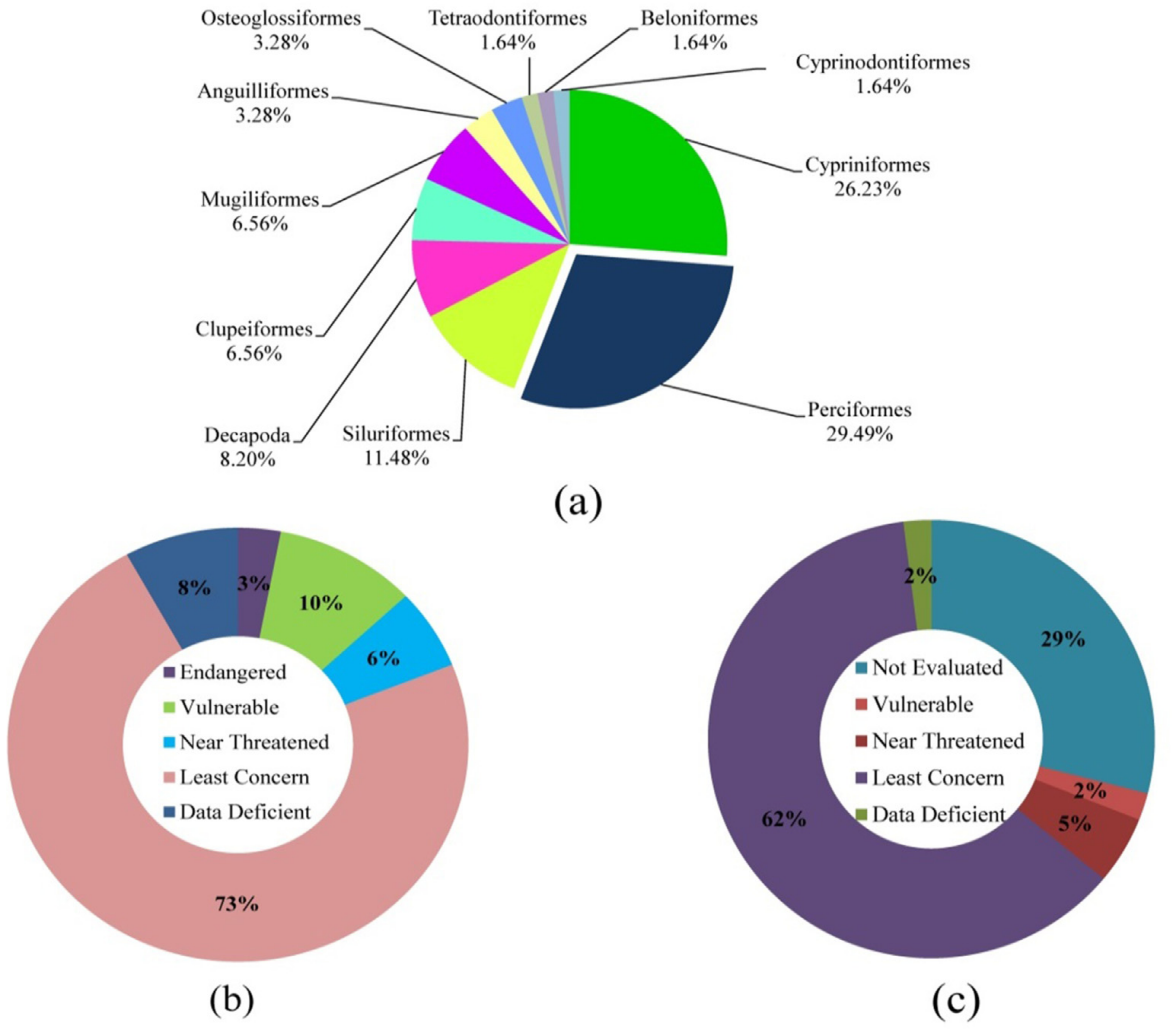
**(a)** Order-based fish species available in the study area, **(b)** threatened fish availability according to IUCN status (Bangladesh, 2015), and **(c)** IUCN status (global, 2015).

As a coastal river, the Payra supports a diverse range of fish species belonging to many orders and families. In the fishing region, about 61 species were reported, including 56 finfish and five shellfish species, divided into 22 families and 11 orders. The order basis of fish species availability showed that Perciformes (29.49%) was the dominant order followed by Cypriniformes (26.23%), Siluriformes (11.48%), Decapoda (8.20%), Clupeiformes (6.56%), Mugiliformes (6.56%), Anguilliformes (3.28%), Osteoglossiformes (3.28%), Tetraodontiformes (1.64%), Beloniformes (1.64%) and Cyprinodontiformes (1.64%). Tetraodontiformes, Belonifirmes, and Cyprinodontiformes are the least ordered and found in the least amount in contrast to other families, according to this study (Figure 4a). Cyprinidae was the most abundant family throughout the research period, with 15 species, followed by Gobiidae, Channidae, Mugilidae, Bagridae, Clupeidae, Schilbeidae, and Notopteridae. According to IUCN Bangladesh (2015) among the 61 finfish and shellfish species 4 endangered, 6 vulnerable, 4 near threatened, 42 least concern, and 5 data deficient species were found (Figure 4b). We also recorded 19 species that have yet to be assessed for conservation status by the International Union for Conservation of Nature (IUCN) (Figure 4c). During the study, a total of 61 fish species were found in the Payra river (Table 2) while Hossain et al. (2012) reported 53 species in the Meghna river estuary, Mohshin and Haque (2009) reported 56 fish species in the Mahananda river, Chakraborty and Mirza (2010) reported 66 fish species in the Someswari river, Tongnunui et al. (2016) found 79 species in Mae Klong river, and Islam et al. (2015) enlisted 52 species in Payra river. Our investigation showed more or less identical results for species distribution based on all of these data. Long-term changes in hydrological and climatic conditions are to blame for the loss of species diversity. Another factor for poor species diversity is fishermen's long-term usage of ESBN nets, which catch everything from tiny aquatic invertebrates to giant fish. Payra river, as a coastal river, has a considerable fishery variety and is one of Bangladesh's most species-rich coastal rivers, and it requires good management to re-establish its prior species richness.

**Table 2:** List of fish species found in Payra river (Pandov point) and their contribution with IUCN status IUCN Bangladesh (2015).

Order	Family	Local Name	Common Name	Scientific Name	IUCN (BD)	IUCN (Global)	% Contribution
**Cypriniformes**	Cyprinidae	Catla	Catla	*Gibelion catla* (Hamilton, 1822)	LC	NE	0.1
	Cyprinidae	Mrigal	Mrigal carp	*Cirrhinus cirrhosus* (Bloch, 1795)	NT	VU	0.14
	Cyprinidae	Rui	Roho labeo	*Labeo rohita* (Hamilton, 1822)	LC	LC	0.09
	Cyprinidae	Kalibaus	Black rohu	*Labeo calbasu* (Hamilton, 1822)	LC	LC	0.08
	Cyprinidae	Bata	Bata labeo	*Labeo bata* (Hamilton, 1822)	LC	LC	0.12
	Cyprinidae	Chebli	Giant danio	*D. aequipinnatus* (MacClelland, 1839)	DD	LC	1.09
	Cyprinidae	Darkina	Gangetic scissortail rasbora	*Rasbora rasbora* (Hamilton, 1822)	NT	LC	2.8
	Cyprinidae	Dhela	Cotio	*Osteobrama cotio* (Hamilton, 1822)	NT	LC	1.38
	Cyprinidae	Mola	Indian carplet	*A. microlepis* (De Filippi, 1863)	LC	NE	1.76
	Cyprinidae	Chep chela	Silver hatchet barb	*Chela cachius* (Hamilton, 1822)	VU	LC	1.36
	Cyprinidae	Mola	Mola carplet	*A. mola* (Hamilton, 1822)	LC	LC	1.81
	Cyprinidae	Punti	Puntio barb	*Puntius puntio* (Hamilton, 1822)	DD	NE	1.04
	Cyprinidae	Bhadi punti	Pool barb	*Puntius sophore* (Hamilton, 1822)	LC	LC	5.26
	Cyprinidae	Tit punti	Ticto barb	*Pethia ticto* (Hamilton, 1822)	VU	LC	1.3
	Cyprinidae	Chela	Large razorbelly minnow	*Salmophasia bacaila* (Hamilton, 1822)	LC	LC	2.32
	Cobitidae	Gutum	Guntea loach	*L. guntea* (Hamilton, 1822)	LC	LC	0.8
**Perciformes**	Channidae	Cheng	Dwarf snakehead	*Channa gachua* (Hamilton, 1822)	LC	LC	0.54
	Channidae	Gozar	Great snakehead	*Channa marulius* (Hamilton, 1822)	EN	LC	0.06
	Channidae	Ranga	Walking snakehead	*Channa orientalis* (Block & Schneider, 1801)	LC	LC	0.47
	Channidae	Taki	Spotted snakehead	*Channa punctata* (Bloch, 1793)	LC	LC	0.45
	Channidae	Shol	Striped snakehead	*Channa striata* (Bloch, 1793)	LC	LC	0.3
	Anabantidae	Koi	Climbing perch	*Anabas testudineus* (Bloch, 1792)	LC	DD	0.54
	Ambassidae	Nalua chanda	Scalloped perchlet	*Ambassis nalua* (Hamilton, 1822)	DD	LC	1.8
	Ambassidae	Ranga chanda	Indian glassy fish	*Parambassis ranga* (Hamilton, 1822)	LC	NE	1.4
	Gobiidae	Bele	Tank goby	*Glossogobius giuris* (Hamilton, 1822)	LC	LC	1.21
	Gobiidae	Bailla	Pacific river goby	*Awaous guamensis* (Valenciennes, 1837)	LC	LC	0.65
	Gobiidae	Chewa	Pointed-tailed goby	*Psedapocryptes elongates* (Cuvier, 1837)	LC	LC	1.56
	Gobiidae	Dogri	Burrowing goby	*Trypauchen vagina* (Bloch & J.G. Schneider, 1801)	LC	NE	1.35
	Gobiidae	Dali chewa	Mudskipper	*Apocryptes bato* (Hamilton, 1822)	LC	LC	1.53
	Gobiidae	Dahuk	Walking goby	*S. histophorus* (Valenciennes, 1837)	LC	NE	1.89
	Osphronemidae	Lal kholisha	Dwarf gourami	*Trichogaster lalius* (Hamilton, 1822)	LC	LC	2.07
	Sciaenidae	Poa	Pama croaker	*Otolithoides pama* (Hamilton, 1822)	LC	NE	0.3
	Sillaginidae	Tular dandi	Flathead sillago	*Sillaginopsis panijus* (Hamilton, 1822)	LC	NE	0.31
	Polynemidae	Taposi	Paradise threadfin	*P. paradiseus* (Linnaeus, 1758)	LC	NE	0.8
**Siluriformes**	Bagridae	Gulsha Tengra	Bleeker's mystus	*Mystus bleekeri* (Hamilton, 1822)	LC	LC	1.7
	Bagridae	Tengra	Striped drawf catfish	*Mystus vittatus* (Bloch, 1794)	LC	LC	2.01
	Bagridae	Nuna tengra	Long whiskers catfish	*Mystus gulio* (Hamilton, 1822)	NT	LC	0.84
	Pangasiidae	Pangas	Yellowtail Catfish	*Pangasius pangasius* (Hamilton, 1822)	EN	LC	0.08
	Schilbeidae	Batasi	Indian potasi	*P. atherinoides* (Bloch, 1794)	LC	LC	2.04
	Schilbeidae	Shilong	Silond catfish	*Silonia silondia* (Hamilton, 1822)	LC	LC	0.15
	Schilbeidae	Ghaura	Garua bacha	*Clupisoma garua* (Hamilton, 1822)	EN	NE	1.31
**Clupeiformes**	Engraulidae	Phasa	Gangetic hairfin anchovy	*Setipinna phasa* (Hamilton, 1822)	LC	LC	1.17
	Clupeidae	Chapila	Indian river shad	*Gudusia chapra* (Hamilon, 1822)	VU	LC	1
	Clupeidae	Kachki	Ganges river sprat	*Corica soborna* (Hamilon, 1822)	LC	LC	19.44
	Clupeidae	Ilish	Hilsa shad	*Tenualosa ilisha* (Hamilton, 1822)	LC	LC	0.12
**Mugiliformes**	Mugilidae	Khorsula	Corsula mullet	*Rhinomugil corsula* (Hamilton, 1822)	LC	LC	0.62
	Mugilidae	Bata	Greenback mullet	*Chelon subviridis* (Valenciennes, 1836)	LC	NE	0.9
	Mugilidae	Bata	Broad mouthed mullet	*Paramugil parmatus* (Cantor, 1849)	LC	NE	0.6
	Mugilidae	Parse	Goldspot mullet	*Chelon parsia* (Hamilton, 1822)	LC	NE	0.66
**Osteoglossiformes**	Notopteridae	Chital	Humped featherback	*Chitala chitala* (Hamilton, 1822)	EN	NT	0.09
	Notopteridae	Foli	Bronze featherback	*Notopterus notopterus* (Pallas, 1969)	VU	LC	0.16
**Anguilliformes**	Anguillidae	Banehara	Indian mottled eel	*Anguilla bengalensis* (Gray, 1831)	VU	NT	0.28
	Moringuidae	Rata boura	Purple spaghetti eel	*Moringua raitaborua* (Hamilton, 1822)	DD	NE	2.98
**Beloniformes**	Belonidae	Kakila	Freshwater garfish	*Xenentodon cancila* (Hamilton, 1822)	DD	NE	0.7
**Cyprinodontiformes**	Aplocheilidae	Kanpona	Blue panchax	*Aplocheilus panchax* (Hamilton, 1822)	LC	LC	10.42
**Tetraodontiformes**	Actinopterygii	Potka	Ocellated Puffer Fish	*Tetraodon cutcutia* (Hamilton, 1822)	LC	LC	3.63
**Decapoda**	Portunidae	Sataru kakra	Swimmer crab	*P. sanguinolentus* (Herbst, 1783)	VU	NE	1.18
	Portunidae	Zaji kakra	Blue swimmer crab	*Portunus pelagicus* (Linnaeus, 1758)	LC	NE	1.24
	Palaemonidae	G. chingri	Giant freshwater prawn	*M. rosenbergii* (De Man, 1879)	LC	LC	0.56
	Palaemonidae	Gura chingri	Spinder prawn	*M. tenuipes* (Thomas, 1900)	LC	NE	6.46
	Palaemonidae	Goda chingri	Goda river prawn	*M. scabriculum* (Heller, 1862)	LC	NE	1

**N.B.:** EN: endangered, VU: vulnerable, NT: near threatened, LC: least concern and DD: data deficient, NE: not evaluated, R. (Ranga) chanda; N. (Nalua) chanda; G. (Golda) chingri; G. (Gulsha) Tengra.

According to Islam et al. (2015), there were 23 families in the Payra River, which is extremely comparable to our current study. Perciformes was the most prominent family, while Cypriniformes contributed a significant share of the species abundance, according to the order-based composition.

**Figure 5:**
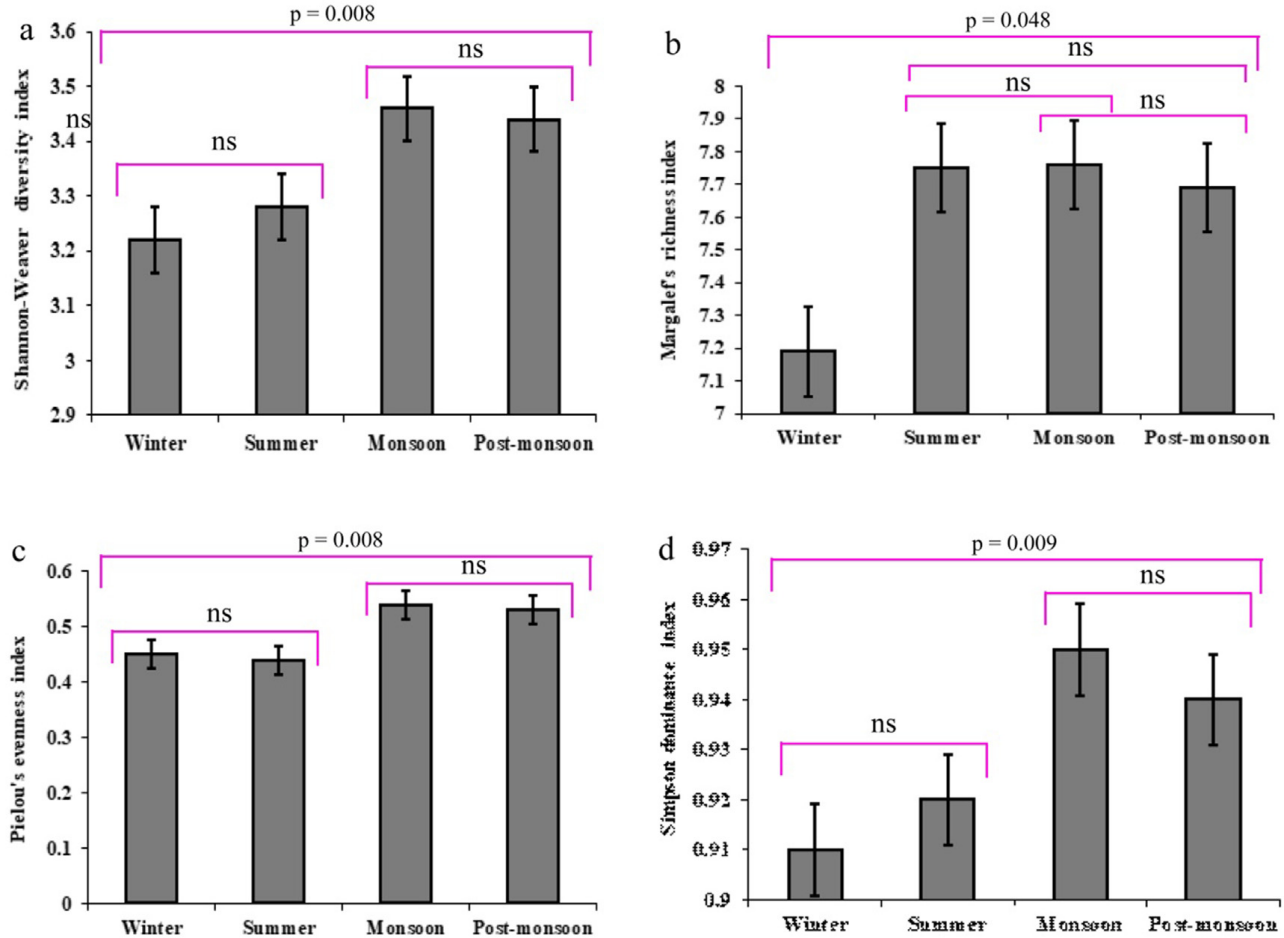
Different diversity indices values were measured in the Payra river, a) Shannon-Weaver diversity index (H'); b) Margalef richness index (d); Pielou's evenness index (J′); and Simpson dominance index (c). Values differed significantly at (*p* < 0.05) among seasons. ‘ns’ indicates no significant difference.

**Table 3:** Shannon-Weaver diversity index and pollution level (Staub et al., 1970).

Value Range (H′)	0–1	1–2	2–3	3–4
**Interpretation**	Heavy	Moderate	Light	Slight
**Present findings (average)**	3.33 ± 0.12			

The goal of a biodiversity index is to use a single number to describe the diversity of a sample or population (Magurran, 1988). The concept of "species diversity" has two parts: the number of species or richness, and the distribution of individuals within species. The Shannon–Weaver diversity index takes into account species diversity, whereas the Evenness and Dominance indices measure the sample's relative number of individuals and the fraction of common species, respectively. The richness and proportion of each species are represented by the Margalef richness index. During the study period, the Shannon-Weaver's diversity value was highest in monsoon (3.46 ± 0.03) and lowest in winter (3.22 ± 0.04) with a mean value of 3.33 ± 0.12 (Figure 5a). This indicates that the study area was most diversified in monsoon and less diversified in the winter. In the Meghna river estuary, Hossain et al. (2012) found 3.19, while Nair et al. (1989) found the same results. For ecological data, the Shannon-Weaver diversity index (H′) value normally varies from 1.5 to 3.5, with a maximum of 5.0 when the samples contain 1,000 species (Hanif et al., 2015). Nabi et al. (2011) reported the Shannon-Weaver value as 0.95–2.62 in the Bakkhali river, In the Kushiyara river, Shannon-Weaver diversity values range from 2.35-2.65, according to Rahman et al. (2018). In each scenario, a low Shannon diversity index is associated with a small number of individuals, while a high Shannon diversity index is associated with a large number of individuals. Seasonal fluctuations in nutrients in seagrass beds, which affect the cohabitation of many fish species (Huh et al., 1985), atmospheric air currents and ambient conditions (Keskin and Ünsal, 1998), and seasonal fish migrations, are the main drivers of differences in biodiversity indexes (Ryer and Orth, 1987). There was a discernible difference in Shannon (H′) diversity. As a result, it is possible to conclude that seasonal differences in species diversity are a typical occurrence in the examined area, indicating a well-distributed fish population in the Payra river. The Margalef richness index (d) value was maximum in monsoon (7.76 ± 0.27) and minimum in winter (7.19 ± 0.38) with an average value of 7.60 ± 0.32 (Figure 5b). Throughout the year-round study period, we can see that from the values the rainy season was the most species-rich season and winter was the least species-rich season. In addition, there was a significant variation in the richness index (d) between seasons (*p* < 0.05). The Margalef richness value, which is employed as a comparison indicator, generally exhibits variation depending on the number of species (Vyas et al., 2012). The highest species number (2234) shows the maximum Margalef richness value (7.91) whereas the minimum value was observed at (6.60) with the lowest number of species (67) (Alam et al., 2013). Vyas et al. (2012) reported the Margalef index in the Betwa River in Madhya Pradesh of India ranging from 3.71 to 6.70. Throughout the year-round study period, we can see that from the values the rainy season was the most species-rich season and winter was the least species-rich season and indicating that the Payra river is usually species-rich.

The Pielou's evenness index (J′) value shows that all the species were more or less evenly distributed all year round but most evenly distributed in monsoon (0.54 ± 0.01) and relatively less in summer (0.44 ± 0.05) with an average value of 0.48 ± 0.05 in the Payra river (Figure 5c). In the case of the evenness index, there was observed fluctuation across seasons (*p* < 0.05). The highest and lowest evenness value recorded in the Halda river was 0.61 and 0.50 respectively (Alam et al., 2013). This means all the species were more or less evenly distributed all year round in the Payra river. The Simpson's dominance index (c) value ranged from 0.95 ± 0.00 in monsoon and 0.91 ± 0.00 in winter with an average value of 0.93 ± 0.02 (Figure 5d). This implies that some species were dominant over others during the winter, but that most species were less dominant during the monsoon in our present study in the Payra river. For the Halda river, the greatest Simpson dominance index value was 0.95, while the lowest value was 0.94, indicating that the dominance was shared by more species for the highest value (Alam et al., 2013) which is fairly in agreement with the present findings. All the findings indicated that the Payra river is a moderately species-rich water body where most of the species were evenly distributed.

### 3.3 Ecological assessment

In the present study, the health condition of the Payra river was found satisfactory according to the pollution level of Staub et al. (1970) based on the Shannon-weaver diversity indices value (Table 3). The pollution level was also assessed by Lad (2015) based on Margalef's Richness index and found clear water (Table 4). We also found four near-threatened species, six vulnerable species, and four endangered species (IUCN Bangladesh, 2015) which may need proper management of this water body to conserve the threatened fishes.

**Table 4:** Margalef's Richness Index (d) and pollution level (Lad, 2015).

Value Range (d)	0–1	1–2	2–4	4–6	>6
**Interpretation**	More Serious Pollution	Serious pollution	Moderate Pollution	Light Pollution	Clear Water
**Present findings (average)**	7.60 ± 0.32				

### 3.4 Cluster analysis

**Figure 6:**
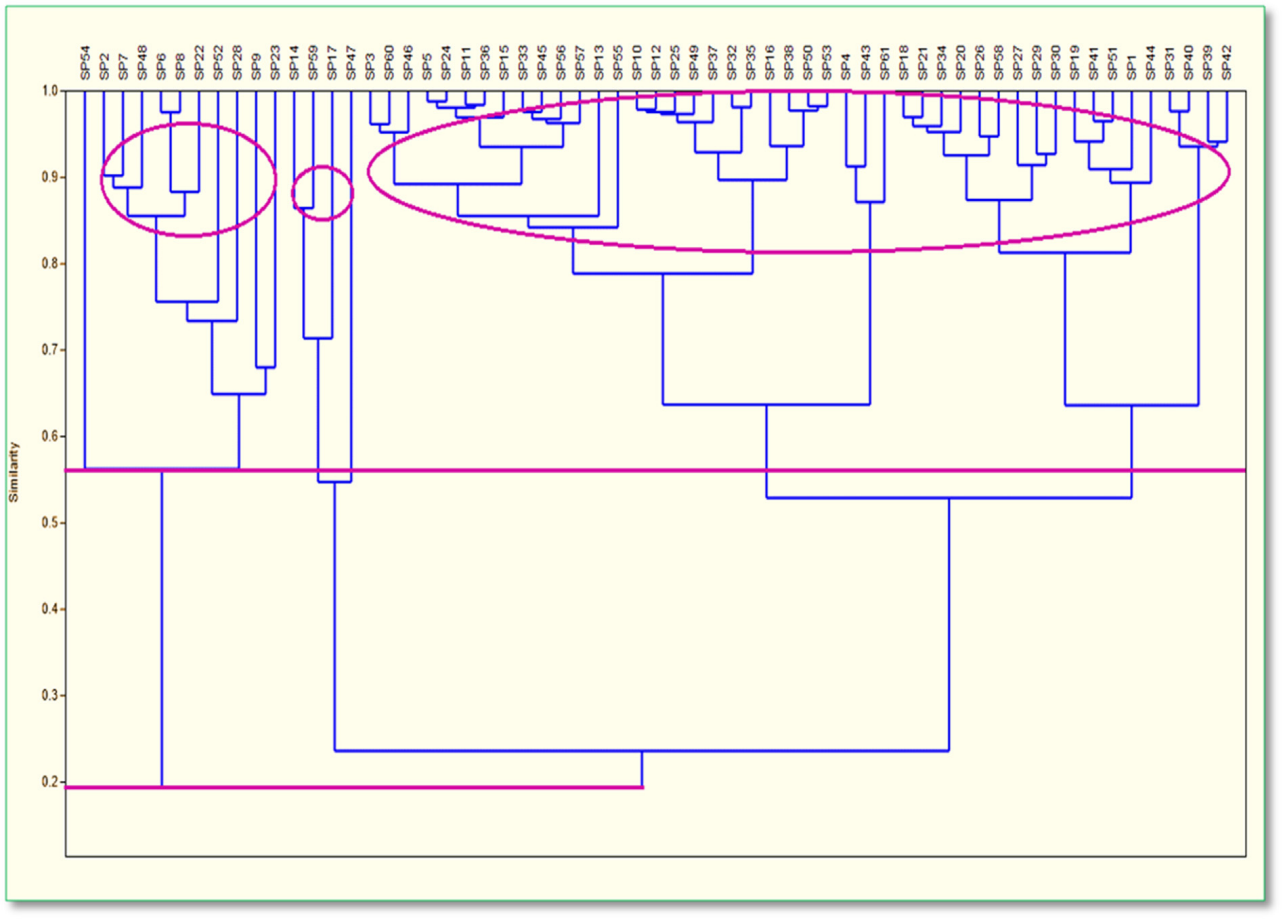
Temporal cluster of fish composition based on Bray–Curtis similarity matrix.

Based on the species encountered, a cluster analysis of the acquired species from the sampling places was performed. According to the Bray–Curtis similarity matrix, the research revealed that the species were 80% similar and were grouped into three clusters. The first cluster, which contains 46 species, was the largest in the Payra river. The second and third clusters, respectively, contain 11 and 4 species. The cluster analysis found a 25% separation for species at the similarity level (Figure 6). First cluster separation starts with *S. silondia* at the similarity level of 25% among all species. Seasonality, which is responsible for fluctuating hydrological and meteorological conditions that change the fish assemblage in estuaries, has a major impact on the similarity and dissimilarity of species occurrence (Loneragan and Potter, 1990; Whitfield, 1989; Young and Potter, 2003). Seasonality has an impact on fish spawning activity, which in turn has an impact on capture composition (McErlean et al., 1973). In the Payra river, we identified 80% similarity among the species and divided them into three distinct clusters at a 25% similarity level. Hossain et al. (2012) discovered more commonalities across months than between various sites, and he revealed two distinct clusters in the Meghna estuary. The presence, distribution, abundance, and diversity of riverine tropical fishes are influenced by several interconnected physical and biological processes. Water salinity, temperature, turbidity, and dissolved oxygen, as well as their regular or irregular variations at various time intervals, have been found as determinants in estuarine fish ecology (Blaber, 2000; Whitfield, 1989). According to Staub's (1970) pollution level based on the Shannon-weaver diversity indices value, the Payra river's health status was judged to be satisfactory in the current study (Table 3). Margalef's Richness index based on Lad (2015) reveals the level of contamination and discovered pure water (Table 4).

### 3.5 Canonical correspondence analysis (CCA)

**Figure 7:**
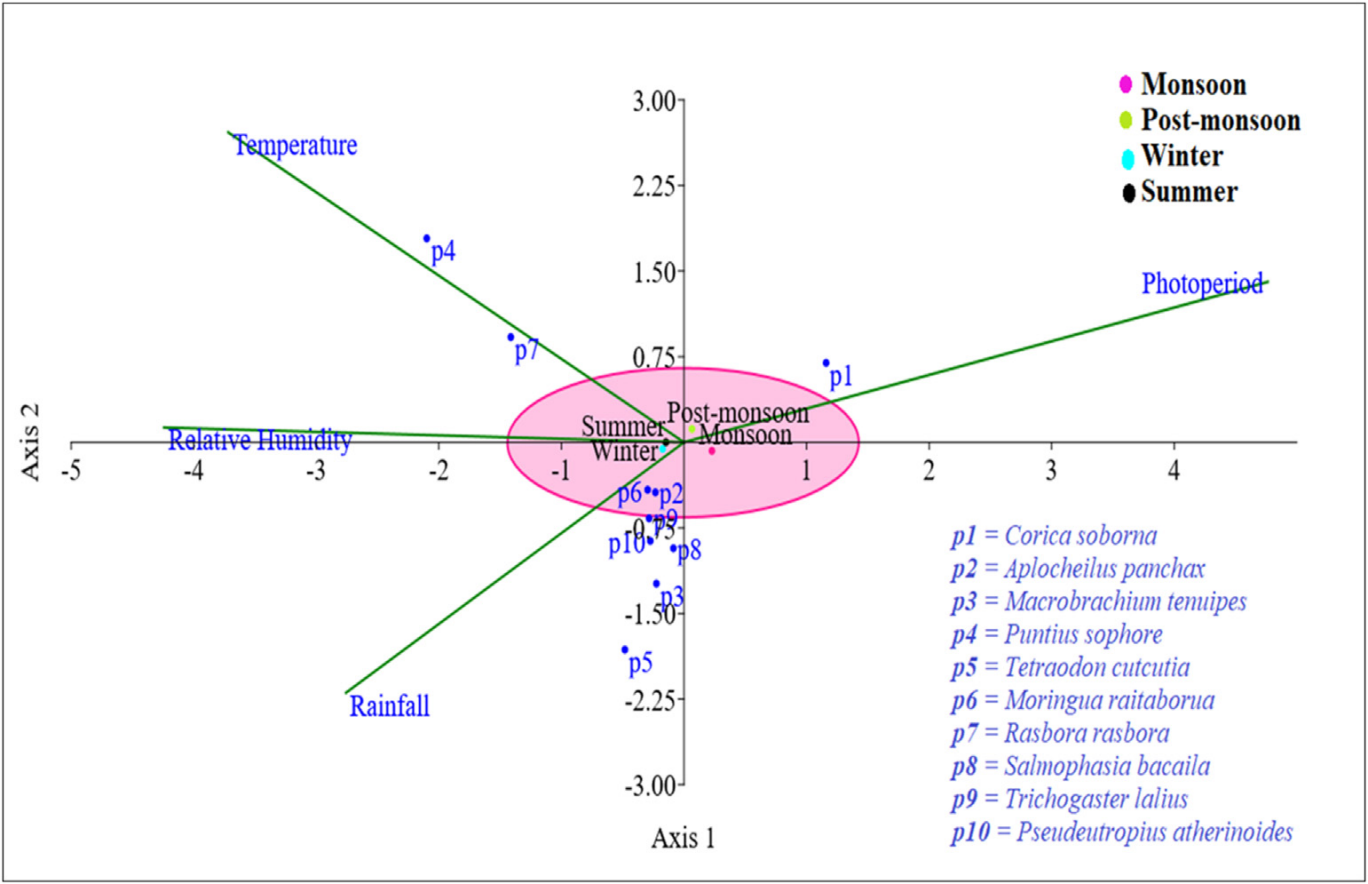
The CCA illustrates the link between climatic variables and the dominant 10 fish species in the study area.

For the first three-axis (CCA1, CCA2, and CCA3), eigenvalues of CCA for meteorological parameters were found to be 0.026616, 0.0059284, and 0.00045777, respectively (Table 5). The cumulative percentage variance of fishery species for the first three axes was 99.997%. The first and second axes constituted 80.65% and 17.96% of the species data, respectively. The results from the first two axes were depicted in Figure 7. Exceptionally the rainfall out of the four meteorological characteristics or variables was found to have a major impact on fish dispersion in the Payra river during different seasons (Figure 7). During the monsoon and post-monsoon seasons, the photoperiod had a significant impact on the *Corica soborna* (p1). On the other hand, *Puntius sophore* (p4) and *Rasbora rasbora* (p7) are the species most impacted by environmental temperature. Except for p1, p4, and p7, rainfall was the most influential climatic condition for the distribution of all species outside of the winter season. The CCA plot clearly shows that rainfall and temperature were the key determinants of species distribution in the Payra river during the current experiment among the four environmental parameters studied. Moreover, fish communities are found to be highly affected by temperature within estuaries (Cyrus and McLean, 1996). The two most important climatic characteristics determining species assemblage structure in the Kushiyara River (Rahman et al., 2018) were air temperature and rainfall which is in conformation with the present study.

**Table 5:** Eigenvalues, percentage of variance, and percentage of cumulative variance for the first three factors of finfish and shellfish samples collected from the Payra river of Bangladesh.

Axes	Eigenvalue	% of Variance	Cumulative %	P value
CCA1	0.026616	80.65	80.65	0.749
CCA2	0.0059284	17.96	98.61	0.08
CCA3	0.00045777	1.387	99.997	0.873

### 3.6 Assessment of the ongoing threats to the biodiversity of the Payra river

**Figure 8:**
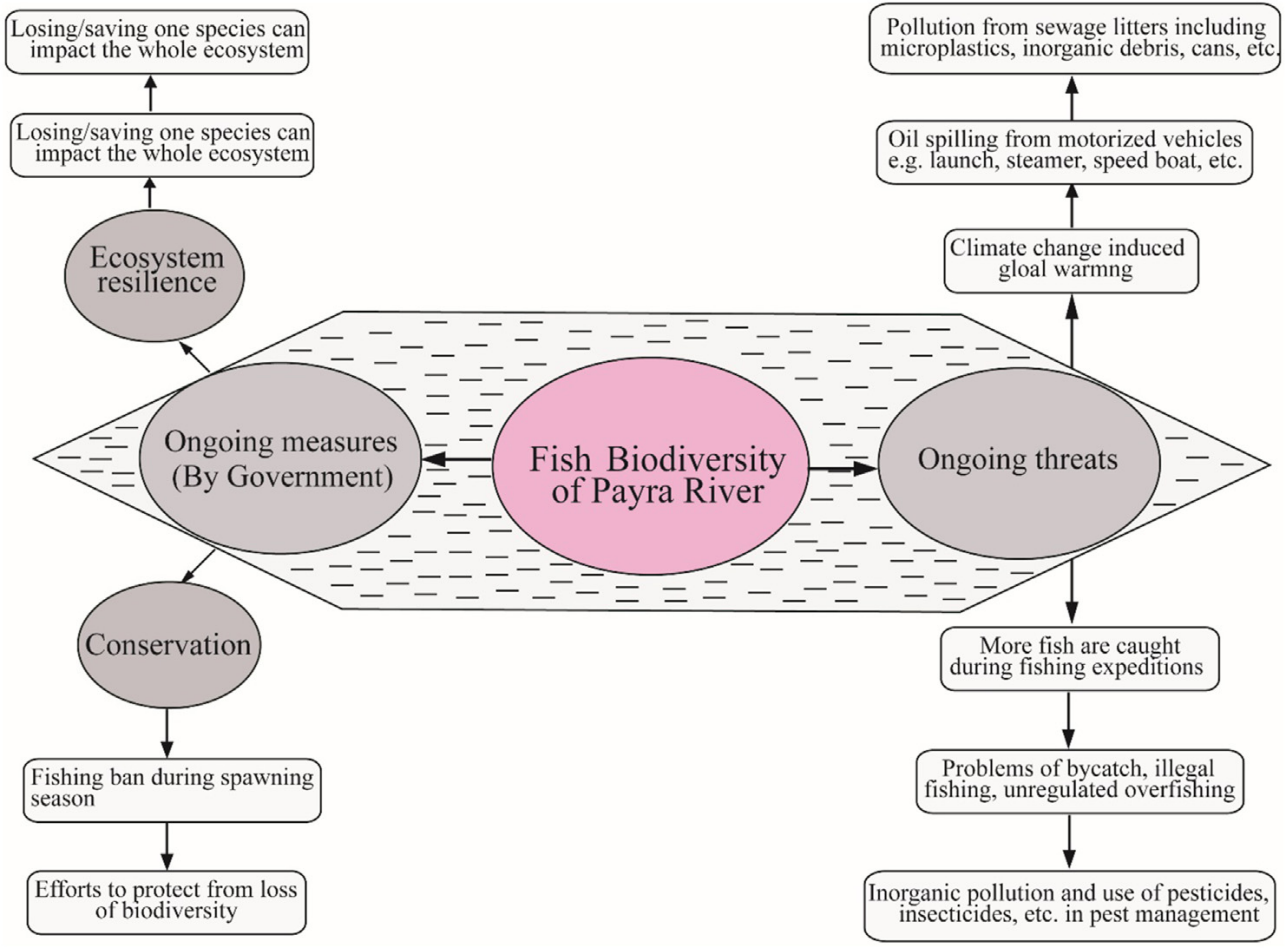
The current state of fish and shellfish biodiversity in the Payra River, as well as the government's ongoing threats mitigation measures.

Wetlands may be disappearing faster than other types of habitats on the planet. In reality, siltation of water bodies adds to the loss and degradation of aquatic habitats (Craig et al., 2004). Rivers carry 2.4 billion metric tons of silt per year, which is deposited on riverbeds, floodplains, and beel bottoms (Spillmann and Bachler, 1993). Man-made activities were discovered to be the leading driver of fish species loss in the Payra river during the research period. Although the government has taken tremendous steps to conserve fish and has legislated a sustainable catch, indiscriminate overfishing persists during the duration of the fishing ban **(**Figure 8). Overfishing is thought to be a major factor in the deterioration of fisheries (Zalinge et al., 2001). Indiscriminate fishing with various non-selective fishing equipment have been identified as a serious hazard to fish decline. Ten varieties of fishing gear were discovered in the Payra River, including five nets (estuarine set bag net, beshal jal, cotton net, behundi net, kachki jal); three hooks and lines (long line, line, hook); and two traps (box trap and chai). Sultana et al. (2016) found 18 different types of fishing gear used by fishermen in the Payra river, including 5 gill nets, 1 seine, 2 fixed purses, 1 lift, 1 cast, 2 push/drag nets, 2 traps, 2 hooks and lines, and 2 wounding gears. During the study period, the amount of behundi net that was destroyed was enormous. In 100 g of behundi net-caught fish, over 200 to 250 individuals of 13 fish species were discovered. According to the present study, cotton nets and behundi nets are causing considerable harm to the Payra river's fish species. Aside from inorganic pollution, pesticides and insecticides used in riverside agriculture along the Payra River, such as hexaconazole and propiconazole, can be deleterious to the fishes. Because hexaconazole produces oxidative stress in fish, and altered mitochondrial bioenergetics may be a reaction to the increased oxidative damage.

**Figure 9:**
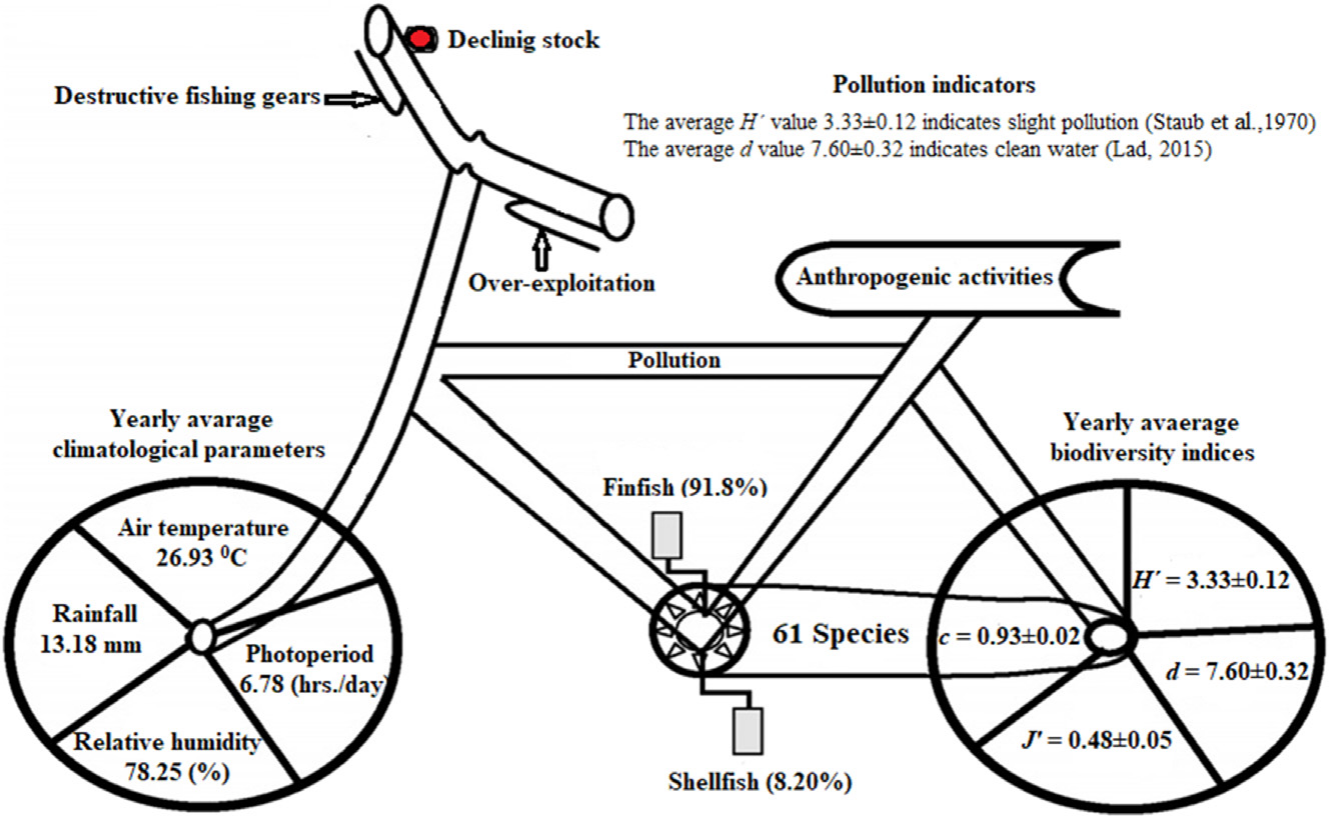
Driving forces of finfish and shellfish temporal distribution at Payra river Bangladesh.

Propiconazole, on the other hand, causes liver damage and significantly slows the growth of zebrafish embryos and larvae after exposure (Figure 9). Several drains on both sides of the river result various chemical wastes from diverse sources damaging the water quality, destructing the spawning and nursing sites of several commercially important Payra river fish species. Pesticides are mostly toxic, making them dangerous to aquatic organisms. They also impair ecosystem integrity and function (Parveen and Faisal, 2002). The installation of diversion canals and sluice gates, on the other hand, generates considerable siltation in the riverbed. Two large islands have formed in the river as a result of siltation: one at the confluence of the Karkhana and Payra rivers, and the other near the Pangasia union. This siltation problem has a significant impact on water flow, which in turn has an impact on the river's overall ecosystem. Scum forms on the surface of the water as a result of oil spilled primarily from motorized vehicles (launch, steamer, and speed boat), preventing light from penetrating. The combined effects of these threats (siltation, habitat loss, conversion of wetland to agricultural fields, overfishing, dewatering during the lean season, poisoning, pesticides and fertilizers, climate change, and so on) could endanger this waterway's whole fishery assets.

## 4 Conclusion

This research was conducted for determining the temporal distribution of finfish and shellfish in the Payra river. Because of moderate pollution from chemical applications in agricultural land and high surface runoff containing pollution substances, as well as the presence of a brickfield alongside the river polluting both air and water, species diversity is lower than in prior comparable studies. Awareness making to the general fishermen through campaigning and training can result in sustainable fishing, avoiding catch of fish larvae, and can ensure controlled fishing during the breeding season. Finally, because fisheries are a renewable resource, they may be protected by implementing sound management strategies with the support of government agencies, non-governmental organizations, and other national and international organizations. The current study of finfish and shellfish distribution through time should be valuable for further ecological assessment and monitoring of the river's quality and its linked water bodies.

## Declarations

### Author contribution statement

Md. Asikur Rahaman Rahat: Performed the experiments; Contributed reagents, materials, analysis tools or data; Wrote the paper. Nitai Roy: Conceived and designed the experiments; Contributed reagents, materials, analysis tools or data. Md. Rased Khan Manon: Contributed reagents, materials, analysis tools or data. Md. Rahamat Ullah: Analyzed and interpreted the data; Wrote the paper. M. Muhsinul Islam: Performed the experiments; Contributed reagents, materials, analysis tools or data. Md. Tareq Rashid; Khandakar Rakibul Hasan: Performed the experiments. Suprakash Chakma: Analyzed and interpreted the data. Md. Arifur Rahman: Conceived and designed the experiments; Performed the experiments; Analyzed and interpreted the data; Contributed reagents, materials, analysis tools or data.

### Funding statement

Nitai Roy was supported by Ministry of Science and Technology (MoST) of Bangladesh under special allocation [SL-66, BS-279].

### Data availability statement

Data included in article/supp. material/referenced in article.

### Declaration of interests statement

The authors declare no conflict of interest.

### Additional information

No additional information is available for this paper.

## Appendix A Supplementary data

Supplementary Material_revised HELIYON-D-22-04717 R2 Supplementary Material_revised HELIYON-D-22-04717 R2
