# Supplementary material for: Temporal distribution of fishery resources in Payra River: relationship with climatological changes, ecological assessment, and threat assessment
Source: Heliyon. 2022 Sep 12;8(9):e10584. doi: 10.1016/j.heliyon.2022.e10584 (PMC9483589; doi:10.1016/j.heliyon.2022.e10584)
Supplement: Supplementary Material_revised HELIYON-D-22-04717 R2 [file mmc1.docx]

1. **Register of checklist of fishermen for monitoring catch and effort data**

**Survey conducted by:**

Location:……………. Distance (from project site):…………… Km

X= Interviewed 0= Interviewed but not fishing

| Sl. # | Fisherman name | Initials/ code | Gear type | Direction (up/ down) | Calendar day (……………... 2013) | | | | | | | | | | | | | | |
| --- | --- | --- | --- | --- | --- | --- | --- | --- | --- | --- | --- | --- | --- | --- | --- | --- | --- | --- | --- |
|  |  |  |  |  | 1 | 2 | 3 | 4 | 5 | 6 | 7 | 8 | 9 | 10 | 11 | 12 | 13 | 14 | 15 |
| 1 |  |  |  |  |  |  |  |  |  |  |  |  |  |  |  |  |  |  |  |
| 2 |  |  |  |  |  |  |  |  |  |  |  |  |  |  |  |  |  |  |  |
| 3 |  |  |  |  |  |  |  |  |  |  |  |  |  |  |  |  |  |  |  |
| 4 |  |  |  |  |  |  |  |  |  |  |  |  |  |  |  |  |  |  |  |
| 5 |  |  |  |  |  |  |  |  |  |  |  |  |  |  |  |  |  |  |  |
| 6 |  |  |  |  |  |  |  |  |  |  |  |  |  |  |  |  |  |  |  |
| 7 |  |  |  |  |  |  |  |  |  |  |  |  |  |  |  |  |  |  |  |
| 8 |  |  |  |  |  |  |  |  |  |  |  |  |  |  |  |  |  |  |  |
| 9 |  |  |  |  |  |  |  |  |  |  |  |  |  |  |  |  |  |  |  |
| 10 |  |  |  |  |  |  |  |  |  |  |  |  |  |  |  |  |  |  |  |
| 11 |  |  |  |  |  |  |  |  |  |  |  |  |  |  |  |  |  |  |  |
| 12 |  |  |  |  |  |  |  |  |  |  |  |  |  |  |  |  |  |  |  |
| 13 |  |  |  |  |  |  |  |  |  |  |  |  |  |  |  |  |  |  |  |
| 14 |  |  |  |  |  |  |  |  |  |  |  |  |  |  |  |  |  |  |  |
| 15 |  |  |  |  |  |  |  |  |  |  |  |  |  |  |  |  |  |  |  |
| 16 |  |  |  |  |  |  |  |  |  |  |  |  |  |  |  |  |  |  |  |
| 17 |  |  |  |  |  |  |  |  |  |  |  |  |  |  |  |  |  |  |  |
| 18 |  |  |  |  |  |  |  |  |  |  |  |  |  |  |  |  |  |  |  |
| 19 |  |  |  |  |  |  |  |  |  |  |  |  |  |  |  |  |  |  |  |
| 20 |  |  |  |  |  |  |  |  |  |  |  |  |  |  |  |  |  |  |  |

1. **Catch and Effort Recording Form**

**Survey conducted by:**

Location:……………. Distance (from project site):…………… Km Gear position:…….. (up/ down stream)

| Name of the fisherman | Hours fishing (since 00.00) (for traps & GN # of hours between last two hauls) | Extra planned hours (or extra soak hours since last trap/ GN until 23.59 midnight) | Fishing effort by gear types | | | | | | Catch (Kg) (since 00.00) | Comments |
| --- | --- | --- | --- | --- | --- | --- | --- | --- | --- | --- |
|  |  |  | Gear type | Size (length x breadth m2) | Mesh (mm) | # of men in team | Gear orientation (up or down stream facing) |  |  |  |
|  |  |  |  |  |  |  |  |  |  |  |
|  |  |  |  |  |  |  |  |  |  |  |
|  |  |  |  |  |  |  |  |  |  |  |
|  |  |  |  |  |  |  |  |  |  |  |
|  |  |  |  |  |  |  |  |  |  |  |
|  |  |  |  |  |  |  |  |  |  |  |
|  |  |  |  |  |  |  |  |  |  |  |
|  |  |  |  |  |  |  |  |  |  |  |
|  |  |  |  |  |  |  |  |  |  |  |
|  |  |  |  |  |  |  |  |  |  |  |
|  |  |  |  |  |  |  |  |  |  |  |
|  |  |  |  |  |  |  |  |  |  |  |
|  |  |  |  |  |  |  |  |  |  |  |
|  |  |  |  |  |  |  |  |  |  |  |

Signature of the data collector: Date: Time of interview:

1. **Species composition by gear type data form**

**Survey conducted by:**

Location:……………. Distance (from project site):…………… Km

| Gear type |  |  | Gear type |  |  | Gear type |  |
| --- | --- | --- | --- | --- | --- | --- | --- |
| Up or down stream |  |  | Up or down stream |  |  | Up or down stream |  |
| FU or FD |  |  | FU or FD |  |  | FU or FD |  |
| Sample Wt (Kg) |  |  | Sample Wt (Kg) |  |  | Sample Wt (Kg) |  |
| Species | % Wt |  | Species | % Wt |  | Species | % Wt |
|  |  |  |  |  |  |  |  |
|  |  |  |  |  |  |  |  |
|  |  |  |  |  |  |  |  |
|  |  |  |  |  |  |  |  |
|  |  |  |  |  |  |  |  |
|  |  |  |  |  |  |  |  |
|  |  |  |  |  |  |  |  |
|  |  |  |  |  |  |  |  |
|  |  |  |  |  |  |  |  |
|  |  |  |  |  |  |  |  |
|  |  |  |  |  |  |  |  |
|  |  |  |  |  |  |  |  |
|  |  |  |  |  |  |  |  |
|  |  |  |  |  |  |  |  |

(N.B.: FU= Facing up stream; FD= Facing down stream)

Signature of the data collector: Date:…………..

1. **Questionnaire for assessments of threats to the biodiversity of Payra river.**

| **#** | **Major threats** | **Yes** | **No** |
| --- | --- | --- | --- |
| **1** | Unprecedented indiscriminate overfishing |  |  |
| **2** | sewage pollution in liters (microplastics, inorganic debris, cans) |  |  |
| **3** | Vehicles with motorized wheels spew oil (launch, steamer, speed boat) |  |  |
| **4** | Fishing equipment used that is harmful to the environment |  |  |
| **5** | Impacts of climate change (irregular rainfall, temperature rise, flash floods) |  |  |
| **6** | Siltation and sedimentation |  |  |
| **7** | Intensification of agricultural farming (riverside agriculture) |  |  |
| **8** | More fish are caught during fishing expeditions |  |  |
| **9** | Construction of infrastructure for communication |  |  |
| **10** | Inorganic pollution and use of pesticides, insecticides, etc. in pest management (name of pesticide) |  |  |
